# Social behavioral analysis of the influence of residential area and grade on pupils’ myopia rate: a cross-sectional survey in Nanjing, China

**DOI:** 10.3389/fmed.2025.1565313

**Published:** 2025-03-19

**Authors:** Yi-jing Xie, Wen Bai, Yun-fan Zhou, Xin Shui, An-yi Ren, Ying Tang, Xuan Zhou, Qin Jiang, Wei-hong Shang

**Affiliations:** ^1^The Affiliated Eye Hospital, Nanjing Medical University, Nanjing, China; ^2^The Fourth School of Clinical Medicine, Nanjing Medical University, Nanjing, China

**Keywords:** pupils, myopia, behaviors, regression analysis, regional, grade

## Abstract

**Objective:**

This study aims to examine the prevalence of myopia among pupils in different residential areas (city center, nearby suburbs, and far suburbs) and grade levels (lower, middle, and upper), analyzing social behaviors and risk factors to guide early intervention.

**Methods:**

A cross-sectional survey was conducted with elementary students across various regions in Nanjing. A total of 2,342 valid questionnaires were collected. Univariate and multivariate logistic regression analyses were used to identify and assess risk factors for myopia and their variations across regions and grade levels.

**Results:**

The survey revealed an overall myopia prevalence of 35.65% (95% CI = 33.71–37.59) among pupils in Nanjing, with the highest rate observed in nearby suburbs (41.34, 95% CI = 38.37–44.31). Myopia prevalence increased with grade level. Multivariate logistic regression analysis identified 8 significant risk factors for myopia, including visual fatigue, frequent eye rubbing, late bedtimes, heavy study burdens, insufficient time spent outdoors, insufficient device breaks, mobile phone use, and infrequent vision checks. Risk factors for myopia varied by region and grade level. Common risk factors across all groups included visual fatigue and infrequent vision checks. City center exhibited unique risk factors, such as late bedtimes and insufficient time spent outdoors. Nearby suburbs displayed unique risk factors, including heavy study burdens and entertainment-oriented electronic device use. Far suburbs displayed a unique risk factor in the prevalent use of mobile phones. Besides, lower grade students showed notably higher entertainment-oriented electronic device use, while upper grade students were more likely to have late bedtimes and insufficient device breaks.

**Conclusion:**

Different social behavioral factors play a key role in the occurrence of myopia among pupils in different regions and grades, and targeted intervention measures need to be developed based on regional characteristics and grade level features.

## 1 Introduction

Myopia is a refractive error primarily caused by irreversible axial elongation, which may increase the risk of complications in adulthood, including retinal detachment, glaucoma, cataracts, and myopic macular degeneration (1). In recent years, urbanization, coupled with a significant rise in educational demands and a marked reduction in outdoor activity, has contributed to a global increase in myopia incidence, particularly among younger populations (2). This trend is especially pronounced in East and Southeast Asia, where the prevalence of axial myopia in younger generations has escalated significantly. Globally, the prevalence varies widely—approximately 3% of schoolchildren in sub-Saharan Africa are affected by myopia, while the prevalence in high school students in certain parts of East and Southeast Asia ranges from 80 to 90% (3). The situation regarding myopia prevention and control among children and adolescents in China remains concerning, with an obvious trend of myopia occurring at increasingly younger ages. As a result, myopia prevention and control in this demographic has become a national priority (4).

Myopia is a complex, multifactorial condition that results from the interaction of various risk factors. However, the rapid rise in myopia prevalence is primarily linked to a combination of genetic predisposition and environmental influences (5). Numerous studies have shown that children with one or both myopic parents are at a higher risk of developing myopia (6–9), though this genetic factor remains uncontrollable. In contrast, socio-behavioral factors—related to an individual’s lifestyle, behaviors, and social environment—are more modifiable and offer opportunities for intervention. Proposed strategies for myopia prevention include increasing outdoor time, reducing near-work activities, and cultivating good eye habits. However, effective prevention and control of childhood myopia require a comprehensive approach, considering factors such as geographic location, age, and individual differences, along with the implementation of integrated interventions to achieve optimal results.

To dynamically assess the visual acuity of children and adolescents aged 6 to 12 years across different regions of Nanjing, and to compare the factors influencing eye health in each area, this study utilizes statistical data on the myopia rates of pupils in Nanjing. A socio-behavioral survey, conducted through questionnaires, was employed to analyze the myopia-related factors of pupils across different residential areas and grade levels. The findings aim to provide a scientific foundation for promoting eye health awareness and guiding eye education initiatives.

## 2 Objects and methods

### 2.1 Participants

The stratified cluster random sampling method was used to divide Nanjing into three regions: city center, nearby suburbs, and far suburbs. One representative elementary school was randomly selected from each region. In June and July of 2024, electronic questionnaires on the vision health of pupils were distributed to these schools. All students underwent a standard visual acuity test conducted by professional ophthalmologists to screen for myopia. To ensure the authenticity and validity of the responses, students were required to complete the questionnaires in the presence of their parents. A total of 2,389 questionnaires were collected, of which 2,342 were deemed valid, resulting in a validity rate of 98.03%. Of the valid responses, 1,057 were from the city center, 1,020 from the nearby suburbs, and 265 from the far suburbs. The survey included 1,125 female and 1,217 male respondents. Informed consent was obtained from all participants before they completed the survey.

### 2.2 Methodology of the survey

#### 2.2.1 Questionnaire development

The “Vision Health Questionnaire for Elementary School Students” was developed based on the myopia control reports from the International Myopia Institute (IMI) published in 2019 (10, 11), as well as relevant studies on myopia risk factors from both domestic and international sources (12–16). The risk factors in the questionnaire were identified based on search terms in PubMed and Web of Science, including: “myopia risk factors,” “myopia in children,” “myopia in adolescents,” “myopia survey,” and “myopia epidemiology.” Under the guidance of experts from the Eye Hospital of Nanjing Medical University, the questionnaire was created and reviewed. To identify and correct any ambiguous or potentially confusing questions, a pilot survey of the questionnaire was conducted on a small sample. The final version of the Questionnaire addressed various factors, including genetics, habits, outdoor activities, electronic device use, and parental behavioral interventions. It included 21 questions related to myopia, as shown in Table 1 (with further details provided in Supplementary Table S1).

**Table 1:** Behavioral questions related to myopia in the questionnaire and the method of assigning values.

Questionnaire		Features	Response
Genetics			
X1	Whether at least one parent has high myopia	Genetic factor	No = 0, Yes = 1
Habits			
X2	Eye discomfort with a sore, dry or aching sensation	Visual fatigue	No = 0, Yes = 1
X3	Whether rub your eyes vigorously when you feel eyestrain	Eye-rubbing behavior	No = 0, Yes = 1
X4	Whether the bedtime is after 10 o’clock	Sleeping behavior	No = 0, Yes = 1
X5	Whether the duration of homework is more than 1 h	Learning burden	No = 0, Yes = 1
Outdoor Activities			
X4	Whether the recess activities are primarily indoors	Location of recess activities	No = 0, Yes = 1
X7	Whether the main activities after school are indoors	Location of after-school programs	No = 0, Yes = 1
X8	Whether the daily average outdoor activity duration is less than 1 h	Time of outdoor activities	No = 0, Yes = 1
Electronic device use			
X9	Whether the age of starting to use electronic products is before six years old	Contact age	No = 0, Yes = 1
X10	Whether the daily average learning electronic screen is used for more than 1 h	Study duration	No = 0, Yes = 1
X11	Whether the daily average entertainment electronic screen is used for more than 1 h	Entertainment duration	No = 0, Yes = 1
X12	Whether the rest frequency when using electronic products is too low (unscientific frequency)	Rest strategy	No = 0, Yes = 1
X13	Whether the main type of electronic products used is mobile phone	Type of use (mobile phone)	No = 0, Yes = 1
X14	Whether the main type of electronic products used is tablet computer	Type of use (computer)	No = 0, Yes = 1
X15	Whether the use of electronic products is mainly for entertainment	Purposes of use	No = 0, Yes = 1
X16	Whether to rely more on electronic products	Dependency	No = 0, Yes = 1
Parental behavioral intervention			
X17	Whether parents do not pay much attention to eye protection knowledge	Parental health awareness	No = 0, Yes = 1
X18	Whether parents ignore their children’s eye health	Parental attention	No = 0, Yes = 1
X19	Whether the way of parent–child companionship is mainly to watch electronic products	Parent–child companionship	No = 0, Yes = 1
X20	Whether parents take their children to places outside the hospital for vision-related examinations	Vision screening locations	No = 0, Yes = 1
X21	Whether parents take their children for an eye exam for more than half a year.	Frequency of vision examinations	No = 0, Yes = 1

#### 2.2.2 Quality control

Before the survey began, a dedicated team was established to refine the questionnaire based on the results of a pre-survey. All questionnaires were completed using real names. During the data collection process, one person was assigned to oversee the questionnaires. Afterward, another individual was responsible for managing the data, ensuring its completeness, and verifying any logical errors.

### 2.3 Statistical analysis

After the data were cleaned and validated, statistical analysis was performed using R Studio software version 4.0.4. Measurement data were expressed as mean ± standard deviation, while count data were presented as percentages (%). The reliability of the questionnaires was assessed using Cronbach’sαcoefficient. The Cochran-Armitage test was used to calculate the trend in numerical variables. The overall data were analyzed using regression analysis to identify behavioral factors associated with myopia. The detailed analyses included: (1) Univariate logistic regression model was constructed using *p*-value <0.1 threshold to identify potential myopia-related factors. (2) Multivariate logistic regression model was constructed using *p*-value <0.05 threshold to identify myopia-related risk factors, with stepwise regression used to select relevant variables. The goodness-of-fit of the model was assessed using the Hosmer-Lemeshow test. (3) Stratified analyses were performed based on the identified variables, considering pupils from different regions and grades. (4) Differences in risk factors across regions were assessed using the chi-square test, while differences across grades were assessed using both the chi-square test and ordered logistic regression analysis.

## 3 Results

### 3.1 Basic information

The reliability test of the questionnaires showed a Cronbach’s *α* coefficient of 0.99, indicating excellent reliability. A total of 2,389 questionnaires were collected, of which 47 were excluded due to being unqualified, resulting in 2,342 valid responses and an effective recovery rate of 98.03%. Among the valid respondents, 1,057 resided in city center, 1,020 in the nearby suburbs, and 265 in the far suburbs. The sample included 1,217 male respondents (51.96%) and 1,125 female respondents (48.04%). In terms of grade distribution, 995 students (42.49%) were in the lower (1st and 2nd) grades, 862 students (36.80%) in the middle (3rd and 4th) grades, and 485 students (20.71%) in the upper (5th and 6th) grades. The results showed that the myopia detection rate among pupils was 35.65%. A statistically significant difference in myopia rates was found across schools in different districts, with the highest rate of 41.34% observed in the nearby suburbs. Additionally, there was a notable variation in myopia rates by grade, with the myopia rate increasing with grade level (Cochran-Armitage test, *p*-value <0.0001), as shown in Table 2.

**Table 2:** Comparison of myopia rates among primary school students in different regions and grades.

Features	Sample size	Myopia cases	Myopia rate (95% CI)	*χ* ^2^	Cramer’s V	*p* value
Region						
city center	1,020	308	30.20 (27.38–33.02)	28.489	0.110	<0.001*
nearby suburbs	1,057	437	41.34 (38.37–44.31)			
far suburbs	265	90	33.96 (28.26–39.66)			
Gender						
boy	1,217	413	33.94 (31.28–36.60)	3.103	0.036	0.078
girl	1,125	422	37.51 (34.68–40.34)			
Grade						
lower	995	164	16.48 (14.17–18.79)	284.190	0.348	<0.001*
middle	862	407	47.22 (43.89–50.55)			
upper	485	264	54.43 (50.00–58.86)			
Total	2,342	835	35.65 (33.71–37.59)			

*Chi-square test, *p* < 0.05.

### 3.2 Analysis of risk factors associated with myopia

Table 3 presents 21 myopia-related behavioral questions analyzed using univariate logistic regression. This analysis identified 13 factors with *p*-value less than 0.1 as potential myopia-related factors. These factors were further examined through multivariate logistic regression, which revealed 8 significant myopia-related risk factors (see Table 3). The results of the Hosmer-Lemeshow test showed that the *p*-value >0.05, indicating a good model fit. Among habits, visual fatigue symptoms, frequent eye rubbing, late bedtimes, and heavy study burdens were significantly associated with myopia. In terms of outdoor activities, insufficient time spent outdoors was linked to myopia. Regarding electronic device use, insufficient device breaks, mobile phone use were significantly related to myopia. Additionally, parental behaviors, such as infrequent vision checkups, were found to be significantly associated with myopia development.

**Table 3:** Univariate and multivariate logistic regression analysis on myopia-related behavioral problems.

Variable	Univariate analysis		Multivariate analysis		
	OR (95% CI)	*p* value	OR (95% CI)	*p* value	VIF
Confounding variable					
Genetic factor	0.98 (0.83–1.16)	0.813	1.00 (0.83–1.19)	0.958	
Habits					
Visual fatigue	3.29 (2.63–4.10)	<0.001†	2.57 (2.03–3.26)	<0.001*	1.063
Eye-rubbing behavior	2.27 (1.71–3.00)	<0.001†	1.73 (1.27–2.34)	<0.001*	1.036
Sleeping behavior	1.79 (1.46–2.21)	<0.001†	1.45 (1.15–1.83)	0.002*	1.098
Learning burden	1.80 (1.52–2.13)	<0.001†	1.29 (1.07–1.56)	0.009*	1.103
Outdoor activities					
Location of recess activities	1.31 (0.99–1.73)	0.057†	1.05 (0.78–1.43)	0.734	
Location of after-school programs	1.47 (1.19–1.80)	<0.001†	1.21 (0.97–1.52)	0.097	
Time of outdoor activities	2.00 (1.57–2.54)	<0.001†	1.67 (1.29–2.16)	<0.001*	1.013
Electronic device use					
Contact age	0.96 (0.81–1.13)	0.603	0.86 (0.72–1.04)	0.122	
Study duration	1.15 (0.91–1.44)	0.249	0.86 (0.65–1.15)	0.311	
Entertainment duration	1.00 (0.79–1.27)	0.973	0.99 (0.73–1.35)	0.953	
Rest strategy	1.50 (1.23–1.83)	<0.001†	1.53 (1.21–1.92)	<0.001*	1.125
Type of use (mobile phone)	1.20 (0.98–1.47)	0.085†	1.63 (1.20–2.20)	0.002*	1.059
Type of use (computer)	1.11 (0.93–1.32)	0.237	1.24 (0.95–1.62)	0.115	
Purposes of use	0.80 (0.67–0.95)	0.011†	0.84 (0.69–1.03)	0.102	1.112
Dependency	1.32 (1.11–1.56)	0.002†	1.17 (0.95–1.43)	0.136	
Parental behavioral intervention					
Parental health awareness	1.11 (0.92–1.34)	0.269	0.93 (0.75–1.15)	0.500	
Parental attention	1.10 (0.62–1.92)	0.749	1.59 (0.85–2.95)	0.144	
Parent–child companionship	1.73 (0.92–3.26)	0.090†	1.22 (0.60–2.46)	0.584	
Vision screening locations	1.03 (0.82–1.30)	0.768	1.05 (0.82–1.36)	0.691	
Frequency of vision examinations	0.37 (0.29–0.46)	<0.001†	0.29 (0.23–0.37)	<0.001*	1.079

†Univariate logistic regression, *p* < 0.1; *Multivariate logistic regression, *p* < 0.05.

### 3.3 Regional myopia-related risk factors among elementary school students

As shown in Table 4, multivariate logistic regression analysis of myopia-related candidate factors in city center, nearby suburbs, and far suburbs revealed 5 common risk factors across all regions. In the city center, these risk factors included visual fatigue symptoms, frequent eye rubbing, late bedtimes, insufficient time spent outdoors, and infrequent vision checkups. In the nearby suburbs, the identified risk factors were visual fatigue, heavy study burdens, insufficient device breaks, entertainment-oriented electronic device use, and infrequent vision checkups. In the far suburbs, the risk factors included visual fatigue, frequent eye rubbing, insufficient device breaks, mobile phone use, and infrequent vision checkups.

**Table 4:** Multivariate logistic regression analysis of risk factors related to myopia in different regions.

Variable	City center		Nearby suburbs		Far suburbs	
	OR (95% CI)	*p* value	OR (95% CI)	*p* value	OR (95% CI)	*p* value
Habits						
Visual fatigue symptoms	2.07 (1.46–2.91)	<0.001*	2.96 (2.05–4.27)	<0.001*	3.48 (1.75–6.94)	<0.001*
Eye-rubbing behavior	2.08 (1.27–3.41)	0.004*	1.38 (0.91–2.09)	0.131	3.70 (1.21–11.34)	0.022*
Sleeping behavior	1.92 (1.34–2.75)	<0.001*	1.35 (0.97–1.86)	0.071	1.30 (0.48–3.53)	0.605
Learning burden	1.09 (0.81–1.49)	0.560	1.37 (1.04–1.82)	0.025*	1.31 (0.70–2.47)	0.397
Outdoor activities						
Location of recess activities	1.15 (0.70–1.89)	0.589	0.90 (0.59–1.38)	0.625	1.34 (0.52–3.42)	0.542
Location of after-school programs	1.40 (0.99–1.99)	0.058	0.96 (0.67–1.37)	0.811	1.19 (0.62–2.29)	0.594
Time of outdoor activities	2.13 (1.42–3.19)	<0.001*	1.41 (0.97–2.06)	0.073	1.19 (0.52–2.74)	0.680
Electronic device use						
Contact age	0.85 (0.64–1.13)	0.270	0.94 (0.72–1.23)	0.651	0.69 (0.36–1.33)	0.265
Rest strategy	1.20 (0.81–1.77)	0.354	1.59 (1.15–2.20)	0.005*	2.65 (1.34–5.24)	0.005*
Type of use (mobile phone)	1.19 (0.76–1.84)	0.444	1.19 (0.85–1.67)	0.306	2.01 (1.14–3.57)	0.017*
Purposes of use	0.89 (0.65–1.23)	0.486	0.73 (0.55–0.96)	0.023*	0.62 (0.34–1.15)	0.133
Dependency	1.08 (0.79–1.48)	0.626	1.10 (0.83–1.47)	0.512	1.63 (0.88–3.01)	0.119
Parental behavioral intervention						
Parent–child companionship	0.52 (0.12–2.24)	0.382	1.72 (0.73–4.07)	0.216	1.30 (0.07–24.57)	0.859
Frequency of vision examinations	0.33 (0.22–0.50)	<0.001*	0.26 (0.19–0.37)	<0.001*	0.29 (0.15–0.54)	<0.001*

*Multivariate logistic regression, *p* < 0.05.

Of particular relevance are the findings that the presence of visual fatigue symptoms and infrequent vision checkups emerged as common myopia-related risk factors across all three regions. Late bedtimes and insufficient outdoor time were unique risk factors identified in city center. Heavy study burdens and entertainment-oriented electronic device use were unique risk factors in the nearby suburbs. Mobile phone use as the primary electronic device was a unique risk factor identified in the far suburbs.

**Figure 1:**
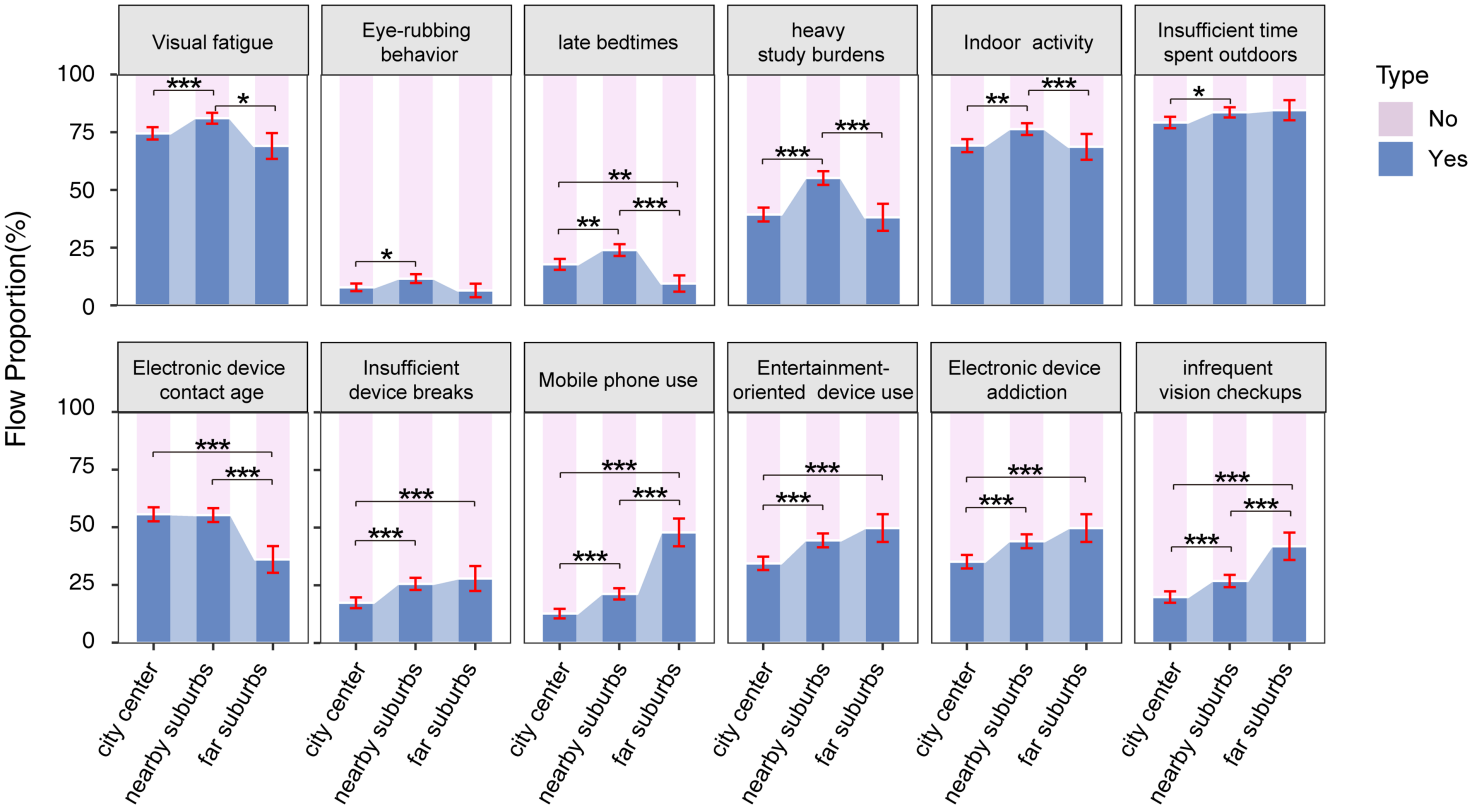
Proportion of distribution of candidate factor questionnaire results related to myopia in different residential areas (city center, nearby suburbs, and far suburbs). The error bars represent 95% confidence intervals. Chi-square test with Bonferroni’s *post hoc* test was used. * *p* < 0.05, ** *p* < 0.01, *** *p* < 0.001.

As shown in Figure 1, results reveal regional differences in myopia-related behaviors among students in city center, nearby suburbs, and far suburbs. In the city center, electronic device use begins at an earlier age. In nearby suburbs, visual fatigue symptoms, frequent eye rubbing, late bedtimes, heavy study burdens, and insufficient time spent outdoors were more common. In contrast, students in far suburbs showed a higher reliance on electronic devices, predominantly for entertainment, with fewer breaks from device use and less frequent vision checkups.

### 3.4 Grade-based myopia-related risk factors among elementary school students

As indicated in Table 5, multivariate logistic regression analysis was conducted on myopia-related candidate factors in the lower (1st and 2nd), middle (3rd and 4th), and upper (5th and 6th) grades, respectively. The analysis identified 5 risk factors for each of the three grade levels specifically, in the lower grades, the identified risk factors included the presence of visual fatigue symptoms, frequent eye rubbing, entertainment-oriented electronic device use, high electronic device addiction, and infrequent vision checkups. In middle grades, the risk factors were symptoms of visual fatigue, frequent eye rubbing, insufficient time spent outdoors, high electronic device addiction, and infrequent vision checkups. In upper grades, symptoms of visual fatigue, late bedtimes, insufficient time spent outdoors, insufficient device breaks, and infrequent vision checkups were observed.

**Table 5:** Multivariate logistic regression analysis of risk factors related to myopia in different grades.

Variable	Lower grades		Middle grades		Upper grades	
	OR (95% CI)	***p*** value	OR (95% CI)	***p*** value	OR (95% CI)	***p*** value
Habits						
Visual fatigue symptoms	2.33 (1.52–3.5)	<0.001*	2.80 (1.92–4.07)	<0.001*	2.48 (1.52–4.05)	<0.001*
Eye-rubbing behavior	1.92 (1.12–3.30)	0.018*	2.10 (1.25–3.51)	0.005*	1.54 (0.78–3.05)	0.213
Sleeping behavior	0.67 (0.37–1.22)	0.191	1.33 (0.94–1.88)	0.112	1.60 (1.02–2.50)	0.039*
Learning burden	1.14 (0.78–1.67)	0.505	0.85 (0.62–1.17)	0.318	0.91 (0.60–1.39)	0.675
Outdoor activities						
Location of recess activities	1.09 (0.62–1.90)	0.765	1.24 (0.74–2.06)	0.411	1.10 (0.59–2.08)	0.760
Location of after-school programs	1.32 (0.87–2.02)	0.195	1.17 (0.81–1.70)	0.401	1.00 (0.60–1.65)	0.989
Time of outdoor activities	1.01 (0.64–1.58)	0.979	2.33 (1.50–3.61)	<0.001*	1.80 (1.09–3.00)	0.023*
Electronic device use						
Contact age	1.31 (0.88–1.96)	0.181	1.24 (0.93–1.67)	0.144	1.37 (0.89–2.09)	0.150
Rest strategy	1.30 (0.81–2.09)	0.282	1.41 (0.97–2.04)	0.072	1.65 (1.07–2.54)	0.024*
Type of use (mobile phone)	1.38 (0.86–2.22)	0.180	1.18 (0.82–1.70)	0.380	1.27 (0.80–2.02)	0.309
Purposes of use	0.65 (0.45–0.94)	0.022*	0.78 (0.57–1.08)	0.133	1.20 (0.78–1.84)	0.418
Dependency	1.45 (1.00–2.09)	0.049*	0.70 (0.52–0.95)	0.024*	0.93 (0.61–1.43)	0.756
Parental behavioral intervention						
Parent–child companionship	0.71 (0.14–3.71)	0.686	1.81 (0.55–6.03)	0.331	0.93 (0.29–2.92)	0.896
Frequency of vision examinations	0.39 (0.24–0.62)	<0.001*	0.30 (0.20–0.45)	<0.001*	0.23 (0.15–0.36)	<0.001*

*Multivariate logistic regression, *p* < 0.05.

A key finding is that visual fatigue symptoms and infrequent vision checkups were prevalent risk factors associated with myopia across all grades. Notably, entertainment-oriented electronic device use emerged as a unique risk factor for lower grades. No unique risk factors were observed in the middle grades. In upper grades, late bedtimes and insufficient device breaks were identified as unique risk factors.

**Figure 2:**
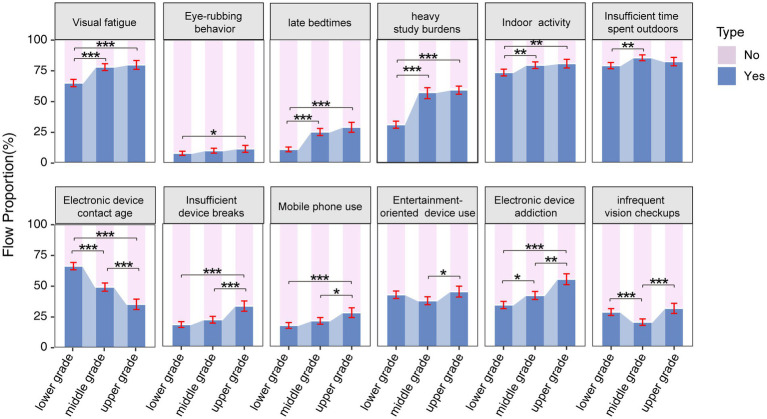
Percentage distribution of the results of candidate factor questionnaire results related to myopia in in the lower (1st and 2nd), middle (3rd and 4th), and upper (5th and 6th) grades. The error bars represent 95% confidence intervals. Chi-square test with Bonferroni’s post hoc test was used. * *p* < 0.05, ** *p* < 0.01, *** *p* < 0.001.

The distribution of myopia-related behaviors across all grades was shown in Figure 2. Ordered logistic regression analysis revealed differences across grade groups on several variables (Table 6). The results indicated a gradual increase in the prevalence of visual fatigue symptoms, eye-rubbing behavior, late bedtimes, heavy study burdens, insufficient device breaks, and mobile phone use as grade levels advance.

**Table 6:** Ordered logistic regression analysis of the effect of grade level on 8 key risk factors related to myopia.

Variable	Middle vs. Lower grades		Upper vs. Lower grades	
	OR (95% CI)	***p*** value	OR (95% CI)	***p*** value
Habits				
Visual fatigue symptoms	1.90 (1.54–2.33)	0.018*	2.10 (1.64–2.73)	0.005*
Eye-rubbing behavior	1.30 (0.94–1.80)	0.107	1.52 (1.05–2.19)	0.023*
Sleeping behavior	2.77 (2.15–3.58)	<0.001*	3.37 (2.55–4.47)	<0.001*
Learning burden	2.87 (2.29–3.60)	<0.001*	3.21 (2.65–3.90)	<0.001*
Outdoor activities				
Time of outdoor activities	1.54 (1.21–1.97)	<0.001*	1.23 (0.94–1.63)	0.142
Electronic device use				
Rest strategy	1.28 (1.01–1.61)	0.035*	2.25 (1.75–2.89)	<0.001*
Type of use (mobile phone)	1.27 (1.01–1.61)	0.043*	1.83 (1.41–2.37)	<0.001*
Parental behavioral intervention				
Frequency of vision examinations	0.63(0.51–0.79)	<0.001*	1.15 (0.90–1.46)	0.234

Lower grades as the reference category; * Ordered logistic regression, *p* < 0.05.

## 4 Discussion

The rapid increase in myopia cases in the 21st century has attracted worldwide attention (17). By 2050, it is projected that 4.758 billion people worldwide will be affected by myopia, with 938 million suffering from high myopia (18). High myopia can lead to pathological changes in various eye structures, including the retina, retinal pigment epithelium, Bruch’s membrane, choroid, optic disk, parapapillary optic nerve area, optic nerve, and sclera, resulting in irreversible vision damage (19). In China, the prevalence of myopia remains high and continues to rise, with an increasing trend in younger age groups. This study examined the prevalence of myopia and its association with social behaviors among pupils across different residential areas and grade levels in Nanjing. We indicate that myopia rates were highest in nearby suburbs and increased with grade level. Additionally, risk factors for myopia varied by area of residence and grade. These results provide a scientific foundation for developing targeted myopia prevention and control strategies for pupils in different regions and grades, aiding in effective prevention and management of myopia.

The development of myopia in children is influenced by multiple factors, including genetic susceptibility (20) and interactions with environmental conditions (21). Our research team has previously conducted in-depth clinical and basic studies on high myopia (22–24). In our study, multivariate logistic regression analysis identified several socio-behavioral risk factors contributing to myopia in children, such as visual fatigue, frequent eye rubbing, late bedtimes, heavy study burdens, insufficient time spent outdoors, insufficient device breaks, entertainment-oriented electronic device use, mobile phone use, and infrequent vision checkups. These risk factors align with findings from previous studies (25). Notably, visual fatigue and frequent eye rubbing are common triggers for myopia, with poor eye habits leading to eye health issues. Excessive academic pressure and limited outdoor activity are two significant environmental factors. As educational competition intensifies, students spend increasing amounts of time on books and homework, and prolonged near-vision tasks inevitably strain the eyes. Simultaneously, urbanization exposes children to electronic devices at younger ages, with increased frequency and duration of use exacerbating visual fatigue, creating a harmful cycle (26).

Several prior studies have reported a higher prevalence of myopia among students in urban areas compared to their rural counterparts (27–29). However, these studies lacked regional specificity. Our research, which included three distinct regions, revealed that myopia detection rates were highest in nearby suburbs. In nearby suburbs, unique risk factors such as heavy study burdens and entertainment-oriented electronic device use were identified. Unlike city centers, nearby suburbs have fewer educational, cultural, and social resources, and parents often have high academic expectations for their children (30). Additionally, the unequal distribution of educational resources, with limited access to quality schools (31), intensifies the competitive pressure on students in these areas, potentially leading to longer study hours, reduced break time. City centers, on the other hand, presented their own set of risk factors, including late bedtimes and insufficient time spent outdoors. The fast-paced lifestyle, nightlife, and late working hours of parents in these areas may influence children’s routines. The widespread use of electronic devices, which occupy much of children’s leisure time, further encourages indoor activities. Moreover, children in city centers often attend tutoring sessions or extracurricular activities after school (32), further limiting their time for outdoor play. Across all three regions, visual fatigue symptoms and infrequent vision checkups were prevalent risk factors. Notably, students in the far suburbs had the lowest frequency of hospital visits for vision exams. Additionally, the use of mobile phones as the primary electronic device emerged as a unique risk factor in the far suburbs. These two factors may be linked to limited awareness of eye health and a lack of emphasis on eye care education in these areas (33). Therefore, educational institutions and families should prioritize regular myopia screenings and the promotion of eye care education (34, 35), particularly in nearby and far suburbs, to raise awareness of visual health and implement targeted interventions.

This study also examined myopia by grade level, revealing significant differences between the lower (1st and 2nd), middle (3rd and 4th), and upper (5th and 6th) grades. Myopia rates increased significantly with grade level, which aligns with findings from previous studies (12, 20, 36, 37). The study identified common myopia-related risk factors across all grade levels, such as visual fatigue and infrequent vision checkups. However, entertainment-oriented electronic device use was a risk factor unique to the lower grades, while short outdoor activity duration, late bedtimes, and fewer breaks from electronic device use were specific to the middle and upper grades. Although no unique risk factors for myopia were identified among middle-grade students, ordinal regression analysis demonstrated that the severity of known risk factors, including visual fatigue symptoms, eye-rubbing behavior, late bedtimes, heavy study burdens, insufficient device breaks, and mobile phone, was intermediate to that observed in lower and upper grades. Increased academic pressures and reduced sleep due to schoolwork have led to more close-up behaviors, particularly prolonged use of electronic devices, which heightens visual fatigue and myopia risk. Additionally, limited extracurricular activities and less time spent outdoors contribute to the accelerated development of myopia.

A comprehensive approach is essential to effectively reduce the prevalence of myopia in elementary school children and protect their visual health. Studies have shown that eye care education in schools and hospitals, along with controlling the amount of close-up screen time, can help reduce visual fatigue (38). Increasing outdoor time can also significantly mitigate the factors contributing to myopia development in school-aged children (39, 40), potentially due to factors like light exposure, peripheral vision, vitamin D levels, and circadian rhythms (41). Sleep deprivation has been linked to myopia progression, particularly with a significant correlation to changes in axial length (42). The American Academy of Sleep Medicine recommends 9 to 12 h of sleep for children aged 6 to 12 years (43). Extended screen time on smart devices has also been associated with an increased risk of myopia (38, 44–46). The American Academy of Pediatrics advises limiting screen time to 1 h of high-quality content per day for children aged 2 to 5 years and setting consistent limits for children aged 6 and older (47). Parents should consciously restrict recreational screen time to help children develop a healthier relationship with their digital devices. To prevent and manage myopia in children, greater emphasis should be placed on improving learning environments, managing study time, and encouraging outdoor activities to ensure adequate eye rest and adjustment.

This study has several limitations. (1) As a single-center, cross-sectional study, the sample size is relatively small, particularly for those in far suburbs. This may limit the representativeness and accuracy of some statistical findings. (2) This cross-sectional study lacks longitudinal data, limiting our ability to draw causal conclusions about the relationship between risk factors and myopia. (3) Additionally, the questionnaire format may introduce information bias due to subjectivity in responses or potential parental influence. Future studies could use more rigorous methods, such as child interviews, to further validate our findings.

## 5 Conclusion

In this study, we conducted an eye health survey among pupils across three regions of Nanjing. Our findings highlight the significant role that various socio-behavioral factors play in the development of myopia among students from different regions and grade levels. This underscores the need for targeted interventions tailored to the specific characteristics of each region and school segment. Myopia results from a combination of factors, and for pupils in the progression stage, it requires a collaborative effort from society, schools, families, and students. Health education and promotion of proper eye care practices are essential for preventing and managing myopia development.

## Data Availability

The raw data supporting the conclusions of this article will be made available by the authors, without undue reservation.
